# Effects of inducible nitric oxide synthase inhibition or norepinephrine on the neurovascular coupling in an endotoxic rat shock model

**DOI:** 10.1186/cc8020

**Published:** 2009-08-26

**Authors:** Bernhard Rosengarten, Stephanie Wolff, Sabine Klatt, Ralf T Schermuly

**Affiliations:** 1 Department of Neurology, Justus Liebig University Giessen, Am Steg 14, 35392 Giessen, Germany; 2 Department of Internal Medicine II, Justus Liebig University Giessen, Klinikstrasse 36, 35392 Giessen, Germany

## Abstract

**Introduction:**

The inducible nitric oxide synthase (iNOS) plays a crucial role in early sepsis-related microcirculatory dysfunction. Compared to a catecholamine therapy we tested effects of a specific iNOS-inhibitor (1400W) on the microcirculatory function in the brain.

**Methods:**

Seventy SD-rats (280-310 g) were divided into 1 control and 6 sepsis groups. Sepsis groups received 1 or 5 mg/kg lipopolysaccharide (LPS) intravenously to induce a moderate or severe sepsis syndrome. Thirty minutes later rats were further randomized into subgroups receiving moderate volume therapy alone or additionally continuous norepinephrine (NE) or 1400W infusion. Separately, effects of 1400W on neurofunctional parameters were investigated in 3 rats without sepsis induction. Performing electric forepaw-stimulation evoked potentials (N2-P1 amplitude, P1-latency) and local hemodynamic responses were recorded with surface electrodes and laser Doppler over the somatosensory cortex at baseline and repeatedly after LPS administration. Cytokine levels (tumor necrosis factor-alpha (TNFα), interleukin-6 (IL6), interferon-gamma (IFNγ)) and cell destruction markers (neuron-specific enolase (NSE), S-100 calcium binding protein B (S100B)) were obtained at the end of experiments.

**Results:**

During sepsis progression resting cerebral blood flow increased and functionally activated hemodynamic responses decreased in a dose-dependent manner. Whereas 1400W and NE improved blood pressure, only 1400W stabilized resting flow levels. However, both regimens were ineffective on the functionally coupled flow responses and destruction markers were similar between groups.

**Conclusions:**

NE and 1400W appeared to be ineffective in mitigating the effects of sepsis on the neurovascular coupling. Other regimens are needed to protect the cerebral microcirculation under septic conditions.

## Introduction

Sepsis and systemic inflammatory response syndromes are the leading causes of mortality in intensive care units [1,2]. Overt nitric oxide (NO) production by the inducible form of NO-synthases (iNOS) is assumed to play an important role in early sepsis-related vasoregulative failure [3,4]. In response to inflammatory stimuli NO levels increase rapidly within minutes to hours [3,4] leading to hypotension [5-7] and refractoriness to vasopressor catecholamines [8]. Animals treated with selective iNOS-inhibitors or transgenic mice deficient in iNOS showed less hypotension and increased microvascular reactivity under septic conditions [9-11].

Regarding the cerebral circulation NO is intimately involved in the adequate blood flow distribution under physiologic conditions [12-14]. The excessive 100- to 1000-fold increase in NO levels overrides the physiologic signals leading to a dissociation of the cerebral circulation. Although the overall perfusion is increased (cerebral hyperemia) [7,15,16] it comes to a dysregulation on the microcirculative level [16,17]. As the brain is very dependent on an appropriate blood supply the microcirculatory failure was in part suggested to best explain the early occurrence of sepsis-associated delirium [17,18].

Whereas catecholamines can restore the macrocirculation there is growing evidence that they do not prevent the occurrence of microcirculatory dysfunction [19] Therefore, inhibition of the iNOS might be an interesting therapeutic regimen in sepsis syndromes. In this study, we compared protective effects of a specific iNOS-inhibitor N-(3-(aminomethyl)benzyl)acetamidine (1400W) with those of norepinephrine (NE) on the cerebral microcirculation as evaluated by the neurovascular coupling mechanism. To make comparison between a moderate or severe sepsis syndrome 1 mg/kg or 5 mg/kg lipopolysaccharide doses were given.

## Materials and methods

### General preparation

All procedures performed on the animals were in strict accordance with the National Institutes of Health Guide for Care and Use of Laboratory Animals and approved by the local Animal Care and Use Committee.

Adult male SD-rats (weighing 280 to 310 g) were initially anesthetized with 1.5 to 3% isoflurane in a 7:3 nitrous oxide (N_2_O)/oxygen mixture of gases, tracheotomized, paralyzed with pancuronium bromide (0.2 mg/kg/h), and artificially ventilated (Harvard Rodent Ventilator; Harvard, South Natick, MA, USA). Arterial blood gas analyses and pH were measured repeatedly as needed and at least every 30 minutes (Blood gas analyzer model Rapidlab 348, Bayer Vital GmbH, Fernwald, Germany). Also, glucose and lactate levels were measured repeatedly (Glukometer Elite XL, Bayer Vital GmbH, Fernwald, Germany; Lactate pro, Arkray Inc. European Office, Düsseldorf, Germany). Glucose was kept in the physiologic range by injections of 0.5 ml 20% glucose as needed. The right femoral artery and vein were cannulated for blood pressure recording, blood sampling, and drug administration. Rectal body temperature was maintained at 37°C using a feedback-controlled heating pad.

The head of the animals was fixed in a stereotaxic frame, the apex of the skull was exposed, and the bone over the left parietal cortex was thinned with a saline-cooled drill to allow transcranial laser-Doppler flowmetry (LDF) [20]. The laser probe (BRL-100, Harvard Apparatus, Holliston, MA, USA) was placed 3.5 mm lateral and 1 mm rostral to the bregma in accordance with the coordinates of the somatosensory cortex; this location corresponds closely to the region of maximal hemodynamic response during contralateral forepaw stimulation [21-23]. The laser-Doppler signal and the systemic mean arterial blood pressure were recorded continuously and processed on a personal computer running a data acquisition software (Neurodyn, HSE, March-Hugstetten, Germany). As the laser Doppler measures flow changes rather than absolute values, resting LDF signals are given in arbitrary units. However, evoked signal changes can be used to assess flow changes and are given in percent-changes from baseline [21,22].

Somatosensory stimulation was carried out with electrical pulses applied by small needle electrodes inserted under the skin of the right forepaw (PSM Module 676, HSE, March-Hugstetten, Germany). Electric brain activity was recorded monopolarily with an active calomel electrode at 0.5 mm behind the laser probe and an indifferent calomel electrode placed on the nasal bone. Signals were recorded and amplified (BPA Module 675, HSE, March-Hugstetten, Germany) and somatosensory evoked potentials (SEP) were averaged using the Neurodyn acquisition software (HSE, March-Hugstetten, Germany). Evoked potential amplitudes were calculated from the N2-P1-amplitude differences and the latency between the start of stimulation and occurrence of the P1-peak was obtained.

Approximately 60 minutes before the stimulation experiments, isoflurane/N_2_O anesthesia was discontinued and replaced by intravenous application of α-chloralose (80 mg/kg; Sigma-Aldrich Chemie GmbH, Taufkirchen, Germany). Supplementary doses of chloralose (30 mg/kg) were given every hour. During chloralose anesthesia, the animals were ventilated with a nitrogen/oxygen mixture of 1/1.

### Neurovascular coupling measurement

Somatosensory activation was carried out by electrical stimulation of the right forepaw with rectangular pulses of 0.3 ms width and a repetition frequency of 2 Hz for 30 seconds. The stimulation current was kept constant at 1.5 mA so that systemic blood pressure changes did not occur [21-23]. Allowing a rest of 30 seconds after each stimulation train, activation-rest cycles were repeated 10 times to increase signal to noise ratio. Flow velocity responses were averaged and relative responses were calculated in relation to the resting phase, setting the resting phase to zero. The evoked flow velocity responses were calculated from the averaged relative flow velocity signals under conditions of stimulation.

### Clinical chemistry

At the end of the experiments blood samples were drawn into tubes containing aprotinin (Trasylol, Bayer AG, Leverkusen, Germany), immediately centrifugated and separated, after which plasma was stored at -80°C until analyses. The neuron-specific enolase (NSE) levels were determined using an ELISA (NSE EIA kit; Hoffmann-La Roche, Basel, Switzerland). The S-100B protein was determined with an immunoluminometric assay (Sangtec 100 LIA; Sangtec Medical, Bromma, Sweden) using monoclonal antibodies specific for the beta subunit of the S-100 protein. Cytokine analysis were performed for IL-6, TNFα, interferon (IFN) γ using commercialized rat ELISA sets (BD Bioscience, Heidelberg, Germany).

### Study design

Each 10 rats were subjected to one of the following groups: control, 1 mg/kg LPS (LPS *Escherichia coli*, O111:B4, Sigma-Aldrich Chemie GmbH, Germany), 5 mg/kg LPS, 1 mg/kg LPS + 1400W, 1 mg/kg LPS + NE, 5 mg/kg LPS + 1400W, 5 mg/kg LPS + NE. LPS was dissolved in 0.5 ml 0.9% sodium chloride (NaCl). LPS was given within two to three minutes. The control group received 0.5 ml vehicle. A moderate volume therapy of 1 to 6 ml/kg/h 0.9% NaCl was allowed in all groups. Thirty minutes after sepsis induction 1400W was given as a bolus of 7.5 mg/kg followed by a continuous infusion at a rate of 7.5 mg/kg/h. NE was given in doses between 0.01 and 10 μg/kg/min to stabilize mean blood pressure in the lower physiologic range between 90 and 100 mmHg.

Prior to and then after LPS administration SEPs, evoked and resting cerebral blood flow velocity levels and blood pressure were measured up to 270 minutes.

In an additional group (n = 3), we investigated the effects of the same dose of 1400W without sepsis induction in healthy rats.

### Statistics

If appropriate, a two-way analysis of variance was performed to assess differences within and between groups. In case of significance a Fischer *post-hoc *test was applied. If assumptions of normal distribution and equality of variances could not be assured, a nonparametric Friedman test was undertaken instead (Statview, SAS, Cary, NA, USA). The significance level was set to *P *< 0.05.

## Results

No rat died from LPS injection. Table 1 shows the group averaged data for partial pressure of carbon dioxide, pH, glucose, lactate, and hemoglobin content. Partial pressure of oxygen levels remained in the range of 240 to 250 mmHg in all groups throughout experiments and therefore were not specified in the table. Table 2 indicates the group data for blood pressure together with the resting LDF signal, N2-P1 potential amplitude, P1 latency, and evoked flow velocity response. The cytokines as well as the cell destruction markers are given in Table 3.

**Table 1:** Group averaged data for glucose, lactate, pH, pCO2 and hemoglobin for all groups

	Glucose (mg/dL)		Lactate (mmol/L)		pH		pCO_2 _(mmHg)		Hemoglobin (mg/l)	
	Base	End	Base	End	Base	End	Base	End	Base	End
**Control**	78 ± 12	82 ± 8	-	0.7 ± 0.7	7.57 ± 0.06	7.53 ± 0.05	32.6 ± 4.5	33.5 ± 2.2	137 ± 7	135 ± 6
**1 mg/kg**	89 ± 18	55 ± 9***	-	2.5 ± 0.8***	7.53 ± 0.05	7.48 ± 0.04*	34.5 ± 5.1	32.8 ± 2.2	137 ± 6	124 ± 15*
**5 mg/kg**	74 ± 19	46 ± 15***	-	2.4 ± 0.7***	7.51 ± 0.05	7.46 ± 0.04***	35.5 ± 4.1	32.3 ± 1.7	133 ± 9	122 ± 15**
**1 mg/kg. + NE**	85 ± 15	68 ± 14*	-	1.4 ± 0.4***	7.52 ± 0.05	7.48 ± 0.05*	35.4 ± 4.0	34.3 ± 3.1	139 ± 10	119 ± 19**
**5 mg/kg + NE**	76 ± 14	70 ± 23	-	1.5 ± 0.6***	7.52 ± 0.03	7.46 ± 0.05**	34.8 ± 3.2	32.8 ± 2.5	145 ± 12	122 ± 17**
**1 mg/kg + 1400W**	78 ± 14	51 ± 7***	-	2.5 ± 0.6***	7.53 ± 0.06	7.46 ± 0.09**	36.4 ± 4.8	35.2 ± 2.6	142 ± 12	121 ± 18**
**5 mg/kg + 1400W**	73 ± 15	46 ± 8***	-	2.4 ± 0.4***	7.52 ± 0.03	7.46 ± 0.04***	34.8 ± 3.4	34.3 ± 1.4	139 ± 9	120 ± 15**

Data are given as mean ± standard deviation (SD) together with statistical results. Significance is given as: * *P *< 0.05; ** *P *< 0.01; *** *P *< 0.001 compared with baseline. 1400W = N-(3-(aminomethyl)benzyl)acetamidine; NE = norepinephrine; pCO_2 _= partial pressure of carbon dioxide.

**Table 2:** Group averaged data for mean blood pressure, somatosensory evoked potentials, P1 latencies, evoked flow velocity responses, and resting LDFV signal, for the different time points of the experiment

		Baseline	30 min	60 min	120 min	180 min	240 min	270 min
**Mean BP (mmHg)**	Control	108 ± 12	108 ± 9	101 ± 12	103 ± 11	104 ± 14	108 ± 18	107 ± 14
**Mean BP (mmHg)**	1 mg/kg	110 ± 11	84 ± 13***	65 ± 8***	66 ± 10***	62 ± 12***	56 ± 9***	63 ± 10***
**Mean BP (mmHg)**	5 mg/kg	115 ± 10	83 ± 17***	62 ± 7***	61 ± 12***	54 ± 14***	50 ± 7***	56 ± 11***
**Mean BP (mmHg)**	1 mg/kg +NE	108 ± 12	84 ± 14***	99 ± 12	92 ± 11	97 ± 11	94 ± 15	95 ± 15
**Mean BP (mmHg)**	5 mg/kg +NE	104 ± 10	79 ± 13***	93 ± 19	90 ± 13	94 ± 12	94 ± 16	92 ± 13
**Mean BP (mmHg)**	1 mg/kg +1400W	106 ± 13	79 ± 18***	80 ± 7**	83 ± 10**	85 ± 10**	79 ± 10***	82 ± 12***
**Mean BP (mmHg)**	5 mg/kg +1400W	110 ± 11	73 ± 16***	77 ± 11**	81 ± 8**	82 ± 10**	78 ± 13***	76 ± 12***
**SEP (μV)**	Control	21 ± 4	20 ± 5	21 ± 5	21 ± 5	20 ± 4	20 ± 4	20 ± 4
**SEP (μV)**	1 mg/kg	21 ± 7	18 ± 6	15 ± 5**	15 ± 4***	12 ± 3***	13 ± 3***	12 ± 4***
**SEP (μV)**	5 mg/kg	20 ± 5	18 ± 5	15 ± 5**	15 ± 5***	13 ± 4***	10 ± 3***	7 ± 2***
**SEP (μV)**	1 mg/kg +NE	21 ± 4	18 ± 4	18 ± 3*	17 ± 3**	16 ± 3**	16 ± 3**	16 ± 3**
**SEP (μV)**	5 mg/kg +NE	21 ± 7	17 ± 4	15 ± 4**	15 ± 4***	13 ± 2***	12 ± 2***	14 ± 3***
**SEP (μV)**	1 mg/kg +1400W	22 ± 3	18 ± 3	11 ± 2***	11 ± 2***	10 ± 2***	9 ± 2***	9 ± 1***
**SEP (μV)**	5 mg/kg +1400W	21 ± 3	17 ± 4	11 ± 2***	11 ± 2***	10 ± 1***	10 ± 2***	10 ± 2***
**P1 latency (ms)**	Control	11 ± 1	11 ± 1	11 ± 2	11 ± 1	12 ± 1	11 ± 1	12 ± 1
**P1 latency (ms)**	1 mg/kg	11 ± 0.4	11 ± 1	12 ± 1*	12 ± 1	12 ± 1	12 ± 2	13 ± 1
**P1 latency (ms)**	5 mg/kg	11 ± 1	12 ± 1	12 ± 1*	12 ± 2	12 ± 1	13 ± 2	13 ± 2
**P1 latency (ms)**	1 mg/kg +NE	12 ± 0.5	12 ± 1	12 ± 1	12 ± 1	12 ± 1	12 ± 1	12 ± 1
**P1 latency (ms)**	5 mg/kg +NE	11 ± 1	12 ± 0.4	12 ± 0.5*	12 ± 2	12 ± 1	12 ± 1	14 ± 3
**P1 latency (ms)**	1 mg/kg +1400W	12 ± 0.5	13 ± 1**	14 ± 1***	14 ± 1***	15 ± 2***	15 ± 3***	15 ± 2**
**P1 latency (ms)**	5 mg/kg +1400W	12 ± 0.5	13 ± 1**	14 ± 1***	14 ± 1***	14 ± 1***	14 ± 1***	15 ± 1*
**EFVR (%)**	Control	20 ± 8	20 ± 6	20 ± 5	16 ± 6	17 ± 5	17 ± 4	18 ± 4
**EFVR (%)**	1 mg/kg	22 ± 10	14 ± 7	10 ± 4**	10 ± 4*	10 ± 4**	11 ± 5**	10 ± 5***
**EFVR (%)**	5 mg/kg	24 ± 7	16 ± 7	7 ± 2***	6 ± 2**	5 ± 2***	6 ± 2***	4 ± 2***
**EFVR (%)**	1 mg/kg +NE	23 ± 7	18 ± 6	16 ± 10	14 ± 10	11 ± 8**	10 ± 7***	9 ± 6***
**EFVR (%)**	5 mg/kg +NE	23 ± 7	14 ± 6	10 ± 7**	5 ± 3***	3 ± 3***	4 ± 3***	5 ± 4***
**EFVR (%)**	1 mg/kg +1400W	18 ± 5	16 ± 8	8 ± 4***	7 ± 3**	6 ± 2***	5 ± 4***	5 ± 3***
**EFVR (%)**	5 mg/kg +1400W	24 ± 7	20 ± 10	7 ± 4***	8 ± 5**	7 ± 5***	6 ± 3***	5 ± 4***
**Resting LDF (U)**	Control	176 ± 49	166 ± 38	170 ± 39	178 ± 33	178 ± 35	183 ± 32	186 ± 32
**Resting LDF (U)**	1 mg/kg	167 ± 60	180 ± 66	185 ± 70	200 ± 95	201 ± 94	203 ± 101	213 ± 110
**Resting LDF (U)**	5 mg/kg	145 ± 25	149 ± 44	132 ± 35	143 ± 37	153 ± 50	166 ± 58	193 ± 84
**Resting LDF (U)**	1 mg/kg +NE	153 ± 57	140 ± 38	187 ± 63	220 ± 96	243 ± 90*	248 ± 92	258 ± 82*
**Resting LDF (U)**	5 mg/kg +NE	162 ± 41	174 ± 57	170 ± 51	208 ± 58	231 ± 55	247 ± 79	267 ± 81*
**Resting LDF (U)**	1 mg/kg +1400W	171 ± 45	156 ± 47	170 ± 90	176 ± 53	185 ± 61	195 ± 63	190 ± 85
**Resting LDF (U)**	5 mg/kg +1400W	141 ± 48	120 ± 40*	128 ± 39	140 ± 58	140 ± 47	141 ± 41	157 ± 47

Data are given as mean ± standard deviation. Statistical results to baseline are given as: * *P *< 0.05, ** *P *< 0.01,*** *P *< 0.001. BP = blood pressure; EFVR = evoked flow velocity responses; LDF = laser-Doppler flowmetry; SEP = somatosensory evoked potentials.

**Table 3:** Data from cytokine and destruction marker measurements as group averaged data ± standard deviation

	NSE ng/l	S-100B ng/ml	IL 6 pg/ml	TNF-α pg/ml	IFN-γ pg/ml
**Control**	0.29 ± 0.14	0.63 ± 0.3	93 ± 28	60 ± 22	32 ± 3
**5 mg/kg LPS**	1.8 ± 0.9	13 ± 8.6	5498 ± 1980	1868 ± 977	1600 ± 540
**5 mg/kg LPS+1400W**	1.6 ± 0.9	11 ± 9.7	4998 ± 1780	1655 ± 877	1800 ± 820
**5 mg/kg LPS +NE**	2.2 ± 0.4	10 ± 7.6	5300 ± 1654	1285 ± 592	1960 ± 660

Compared with control destruction markers and cytokine levels significantly increased in the sepsis groups but did not differ between sepsis groups.

1400W = N-(3-(aminomethyl)benzyl)acetamidine; IFN = interferon; IL = interleukin; LPS = lipopolysaccharide; NE = norepinephrine; NSE = neuron specific enolase.

In non-septic rats, 1400W did not result in changes in the following data: blood pressure (121 ± 11 vs.125 ± 6 mmHg; not significant), glucose levels (60 ± 9 vs.57 ± 6 mmol/L; not significant), resting cerebral blood flow (135 ± 25 vs. 142 ± 28; not significant), evoked flow responses (20 ± 7 vs. 20 ± 8%; not significant), SEP amplitudes (16 ± 6 vs.15 ± 4 μV; not significant), or P1-latencies (10 ± 2 vs.10 ± 1 ms; not significant).

### General findings

With LPS-administration, rats developed signs of a severe sepsis syndrome characterized by a considerable drop in blood pressure (1 mg/kg LPS: 63 ± 10 mmHg; 5 mg/kg LPS: 56 ± 11 mmHg), occurrence of metabolic acidosis (1 mg/kg LPS: 7.48 ± 0.04; 5 mg/kg LPS: 7.46 ± 0.04), and an increase in the lactate levels (1 mg/kg LPS: 2.5 ± 0.8; 5 mg/kg LPS: 2.4 ± 0.7 mmol/l) as indicated in Table 1. Cytokine levels increased in all sepsis groups without differences between groups (Table 3). Resting flow levels were significantly induced in both LPS + NE groups by approximately 50%, whereas 1400W remained neutral (Table 2). Compared with non-treated groups NE improved blood pressure levels effectively in both groups (95 ± 17 mmHg and 92 ± 15 mmHg; both *P *< 0001 to non-treated groups). Not as effective as NE 1400W also stabilized blood pressure levels in both groups (82 ± 12 and 76 ± 12 mmHg; both *P *< 0.001 vs. non-treated animals).

Noteworthy is that only in the 1400W groups glucose substitutions were necessary to maintain adequate blood glucose levels. NE led to even higher glucose levels as compared with the 1 mg/kg or 5 mg/kg LPS groups.

### Neurofunctional findings

**Figure 1:**
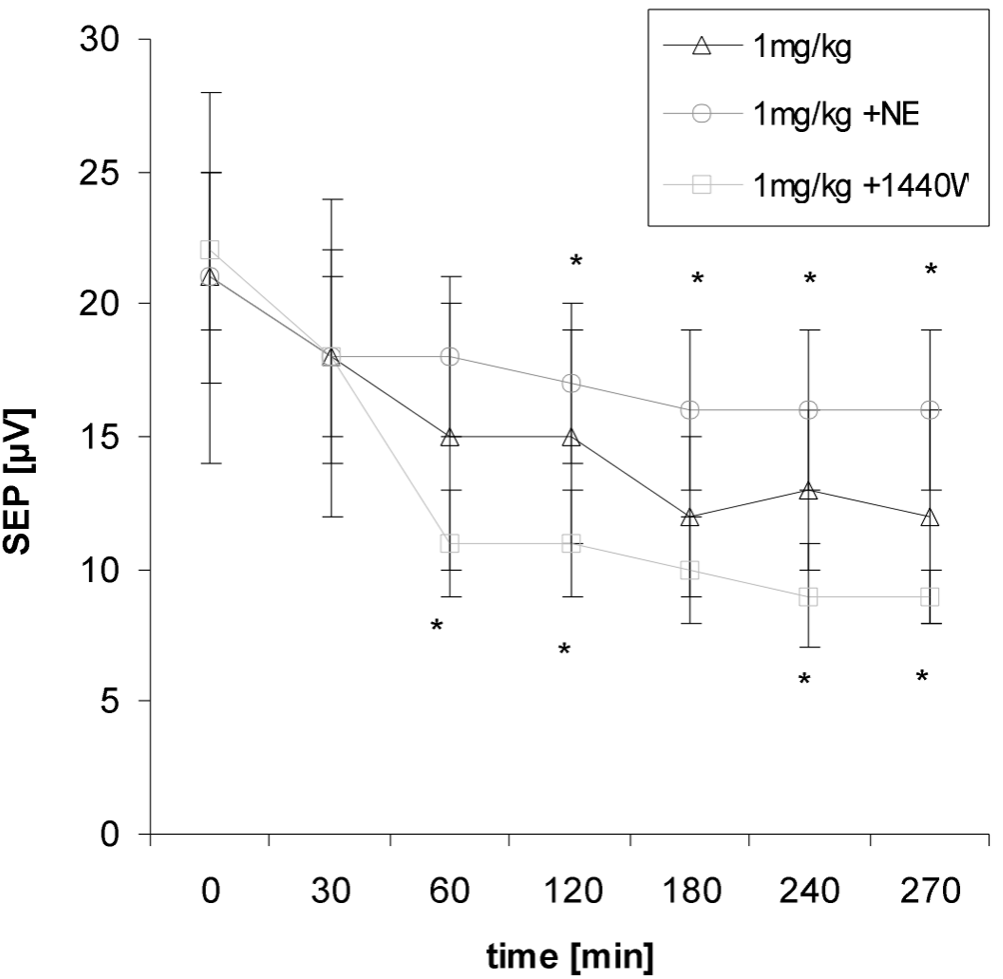
Time course of group averaged N2-P1 amplitudes given as mean ± standard deviation for the 1 mg/kg lipopolysaccharide groups.  Norepinephrine (NE) was protective on the potential amplitudes whereas selective inducible nitric oxide synthase (iNOS)-inhibition (N-(3-(aminomethyl)benzyl)acetamidine (1400W)) showed adverse effects. Statistical results are given as compared to the non-treated group; * *P *< 0.05. SEP = somatosensory evoked potentials.

**Figure 2:**
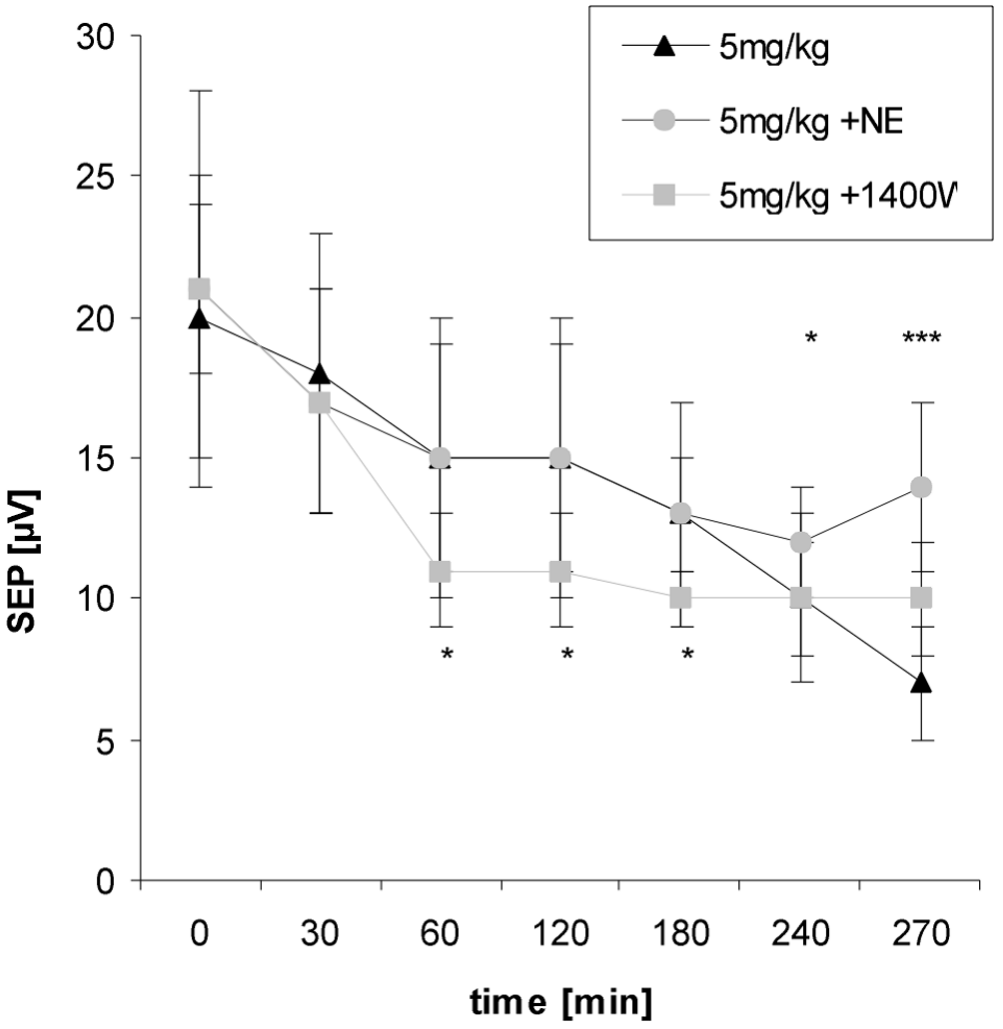
Time course of group averaged N2-P1 amplitudes given as mean ± standard deviation for the 5 mg/kg lipopolysaccharide groups.  Norepinephrine (NE) was protective on the potential amplitudes at the end of experiments whereas selective inducible nitric oxide synthase (iNOS)-inhibition (N-(3-(aminomethyl)benzyl)acetamidine (1400W)) showed again adverse effects from beginning of therapy. Sttistical results are given as compared with the non-treated group; * *P *< 0.05; *** *P *< 0.001.

Addressing neurofunctional parameters NE was shown to be most effective on N2-P1 amplitudes. Compared with the 1 mg/kg LPS group, evoked potential amplitudes were significantly higher throughout experiments (270 minutes: 16 ± 3 μV vs. 12 ± 4 μV; *P *< 0.05; Figure 1). In the 5 mg/kg group, NE prevented a progressive decline of amplitudes at the end of experiments (270 minutes: 14 ± 3 μV vs. 7 ± 2 μV; *P *< 0.001; Figure 2). No protective effects were seen on the P1-latencies or evoked flow velocity responses (Table 2).

1400W led to an early and progressive decline in evoked potential amplitudes, which exceeded changes seen in the 1 mg/kg or 5 mg/kg LPS groups (Figures 1 and 2). Similarly, P1-latencies increased to a higher extent as expected from non-treated LPS groups (Table 2). Evoked flow velocity responses dropped in relation to the decrease in evoked potential amplitudes indicating still intact coupling. This makes the possibility of an artifact in electrical recordings unlikely.

## Discussion

The functionally coupled blood flow responses are decreased during early phases of sepsis, which could contribute to brain dysfunction (sepsis-associated delirium, septic encephalopathy) in sepsis. Neither 1400W nor NE improved the neurovascular coupling. However, interpretation of the effects of the iNOS-inhibition on the neurovascular coupling is hampered by the direct adverse effects of 1400W on SEP, which were only seen under septic conditions. In both LPS groups, potential amplitudes declined and latencies increased directly after administration of the iNOS-inhibitor. In non-septic rats neither 1400W nor other unspecific NO-inhibitors showed this effect [24,25]. A second new finding was the strong glucose lowering effect of 1400W under septic conditions. From the literature a beneficial effect was anticipated because increased NO levels adversely interfere with the mitochondrial function and the intracellular glucose homeostasis [26,27]. However, occurrence of mitochondrial respiratory chain enzyme dysfunction was shown to occur at later stages beginning six to eight hours after sepsis induction [28,29]. Due to a tight glucose control in the present study simple hypoglycemia cannot explain our findings.

**Figure 3:**
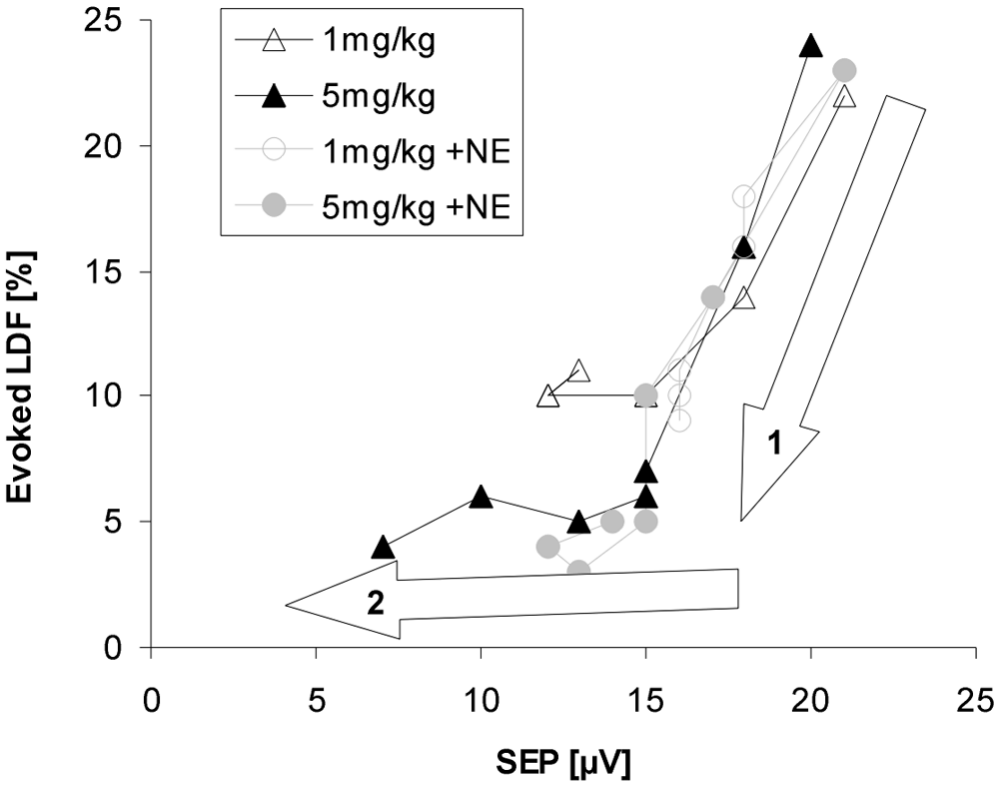
Graph of group averaged evoked potential amplitudes and evoked flow velocity responses to illustrate the temporal aspects of neurovascular dysfunction.  With lipopolysaccharide (LPS) application it comes first to a disproportional high decline in evoked laser-Doppler responses in both LPS dose groups (arrow 1) before somatosensory evoked potential amplitudes declined (arrow 2). This constellation indicates early microcirculatory failure in the septic brain. Whereas norepinephrine (NE) did not modify the drop in hemodynamic responses (arrow 1) it was protective on the evoked potential amplitudes (absent (1 mg/kg) or diminished (5 mg/kg) component of arrow 2). LDF = laser-Doppler flowmetry; SEP = somatosensory evoked potentials.

The effect of NE on evoked potential amplitudes cannot be taken as a clear indication for neuroprotection. An improvement of amplitudes is a direct effect of NE, which has been described even under non-septic conditions [30]. It was explained by a more focused activation of cortical neuronal fields. In line with this interpretation, the oxidative metabolism of the brain did not change in septic patients under NE treatment [31]. The lack of an effect on the cell destruction markers also points against a significant neuroprotective effect. Regarding the cerebral circulation, NE is neutral as long as the blood-brain barrier is intact and exerts vasoconstrictive effects in case of a barrier leakage [32,33]. Therefore, the induced cerebral blood flow in the NE group could be best explained by the higher blood pressure levels. We also did not find an effect of NE on the neurovascular coupling. This is shown in Figure 3 which illustrates the relation between evoked potential amplitudes (x-axis) and resultant flow velocity changes (y-axis) from the beginning to end of experiments. Arrow 1 shows the typical initial uncoupling with a drop in evoked flow velocity responses but still intact evoked potential amplitudes. This response was not modified by NE as compared with non-treated groups. Arrow 2 shows the succeeding drop in evoked potential amplitudes, which were prevented in the NE group possibly due to a substance effect. The typical pattern of an initial uncoupling and succeeding drop in potentials was more pronounced in the 5 mg/kg groups as compared with the 1 mg/kg groups.

Chloralose is a narcotic agent which allows neurophysiologic monitoring [34]. It results in a mild alkalosis which explains the higher initial pH levels.

We chose a classic catecholamine therapy, although immunomodulatory effects of some catecholamines were reported in the literature [35,36]. We did not find a significant effect of NE on the cytokine level and also did not find significant effects on the gene expression levels of chemokines [37]. Our data are therefore in line with others who found epinephrine but not NE to modulate cytokine levels in a porcine model of endotoxic shock [38].

The cell destruction markers NSE and S-100 calcium binding protein B have been widely used to assess the prognosis and outcome of different disease processes [39-41]. A limitation occurs in case of a blood-brain barrier breakdown because serum levels then can considerably vary between individuals. A similar study excluded a blood-brain barrier breakdown for the first hours of a sepsis syndrome [33]. This is reflected by the narrow standard deviation of cell destruction markers.

## Conclusions

Neurovascular coupling is decreased during early phases of sepsis. This could contribute to brain dysfunction in sepsis (sepsis-associated delirium). Neither NE nor 1400W considerably prevented the breakdown of the neurovascular coupling. However, further research is needed to clarify the direct adverse effects of 1400W on neuronal function, which occurs only under septic conditions.

## Key messages

• Microcirculatory dysfunction occurs early in the septic brain.

• Besides its effects on blood pressure, norepinephrine does not prevent the occurrence of sepsis-related cerebral microcirculatory failure and the effect on evoked potential amplitudes seems to be a side effect of the agent.

• Under septic conditions, 1400W stabilizes the blood pressure but shows a direct adverse effect on evoked potential amplitudes which does not appear under physiologic conditions. Due to this effect, interpretation of its effects on the neurovascular coupling is limited; however, a clear beneficial effect was lacking.

## Abbreviations

1400W: N-(3-(aminomethyl)benzyl)acetamidine; ELISA: enzyme-linked immunosorbent assay; IFN: interferon; IL: interleukin; iNOS: inducible nitric oxide synthase; LDF: laser-Doppler flowmetry; LPS: lipopolysaccharide; NaCl: sodium chloride; NE: norepinephrine; NO: nitric oxide; NSE: neuron specific enolase; SEP: somatosensory evoked potentials; TNF: tumor necrosis factor.

## Competing interests

The authors declare that they have no competing interests.

## Authors' contributions

BR drafted the manuscript, performed the experiments together with SK and SW. SW additionally investigated cytokines and cell destruction markers. RS designed the experiments with BR and helped with writing the paper. All authors read and approved the final manuscript.
